# Neonatal acute ethanol intoxication during the epidemic of COVID-19: a case report

**DOI:** 10.1186/s12887-022-03128-1

**Published:** 2022-01-20

**Authors:** Chao Sun, Yanyan Nie, Xiaoyu Cui, Fang Zhang, Yang Liu

**Affiliations:** 1 Department of Neonatology, Tianjin Children’s Hospital/ Tianjin University Children’s Hospital, Tianjin, 300134 P.R. China; 2 Graduate College, Tianjin Medical University, Tianjin, 300070 P.R. China

**Keywords:** COVID-19, Newborn, Acute ethanol intoxication

## Abstract

**Background:**

After the outbreak of COVID-19, many families equip with 75% ethanol to inactivate the SARS-CoV-2, which increases the risk of exposure to ethanol.

**Case presentation:**

We reported a 25-day-old newborn who was diagnosed with neonatal acute ethanol intoxication with a presenting complaint of accidental consumption about 15 ml formula milk containing 75% ethanol. His main clinical manifestations were irritability, flushed skin, tachycardia, tachypnea, and toxicology analysis detected ethanol. After timely gastric lavage and intravenous fluid replacement, he was cured and discharged.

**Conclusions:**

During the COVID-19 epidemic, high concentration ethanol used for inactivating SARS-COV-2 should be placed reasonably and neonatal feeding safety should be emphasized. Timely diagnosis and symptomatic treatment are essential for the prevention and management of acute ethanol intoxication in newborns.

## Background

The major epidemic of COVID-19 has emerged as a serious global public health threat, especially after the emergence of the more infectious Delta strain [1–3]. SARS-COV-2 can be effectively inactivated by 75% ethanol, according to Chinese expert’s consensus [4]. As a result, many homes and public places have been equipped with 75% ethanol for surface disinfection of objects in order to inactivate the SARS-CoV-2, which increases the risk of exposure to ethanol. In 2021 alone, as of September 30, American Association of Poison Control Centers [5] had managed 18,168 ethanol exposure cases about hand sanitizer in children 12 years and younger. However, reports of high concentration of ethanol consumption in children are rare, especially in newborns. Therefore, we reported a case of acute ethanol intoxication in a 25-day-old newborn boy to raise awareness of the disease.

## Case presentation

A 25-day-old male newborn was admitted to Tianjin children’s hospital two hours after accidental consumption of about 15 ml formula milk containing 75% ethanol. According to his mother, two hours before admission, the boy ingested about 15 ml formula milk, which was mistakenly mixed with 75% ethanol instead of water. He immediately developed flushing around his eyes, which gradually spread to his face and the whole body, accompanied by crying and irritability. We reported the incident to the Child Guidance Centre, who conducted a thorough investigation and careful questioning, and ultimately determined that the incident was an unintentional injury.

The boy was delivered by cesarean section at 40 weeks, with the birth weight of 3550 g. His birth history was normal. When he was admitted to the hospital, he was irritated, responsive, cried loudly, and could smell a faint but definite odor of ethanol presenting on the breath. His face and body were flushed. A physical examination showed his body temperature was 37.1 °C, a respiratory rate was 50breaths/min, heart rate was 185beats/min, blood pressure was 83/40 mmHg, and TcSO_2_ was 98%. Besides, his anterior fontanelle was flat and soft, and the examinations of lungs, cardiac, abdominal, nervous system, musculoskeletal, and extremity were normal. His toxicology analysis showed that 4.65 mol/L (21.4 mg/dl) of ethanol was detected in the blood and ethanol was also detected in the gastric juice. The remainder of the examination was normal.

The diagnosis of neonatal acute ethanol intoxication in this child was clear, according to a consensus on the management of acute ethanol intoxication [6]. As a result, we immediately gave normal saline gastric lavage until clear fluid was extracted, intravenous infusion of naloxone, as well as intravenous fluid replacement to maintain internal environment stability, and diuretic and defecation to promote excretion. After 7 h of hospitalization, his facial and body skin flushing completely subsided. 12 h after hospitalization, his mental response returned as usual and he was restarted on formula with good feeding tolerance. After 1 day in hospital, toxicological tests were performed again, and no ethanol was detected in the blood or gastric juices. On the third day of hospitalization, the child was cured and discharged. Periodical follow-up after discharge confirmed that the patient has reached the appropriate age for growth and psychomotor development.

## Discussion and conclusions

Ethanol intoxication is uncommon in children, especially neonates [7]. According to previous reports, the clinical symptoms of acute ethanol intoxication are non-specific, and can be confused with other diseases, such as encephalitis, hydrocephalus, and anaphylactic shock [7, 8].Therefore, we reported a case of acute ethanol intoxication in a 25-day-old newborn boy to raise awareness of the disease.

In this report, we reported an acute ethanol intoxication incident in a 25-day-old newborn who was accidental consumption about 15 ml formula milk containing 75% ethanol during the COVID-19. Unintentional mixing of alcohol in formula is another way for neonates to consume ethanol. In Japan, a case [8] was reported where *sake* (Japanese wine prepared from fermented rice) was mixed in a 15-day-old newborn girl’s formula. The clinical features of the girl were flushed skin, tachycardia and low blood pressure indicating circulatory failure, somnolence and metabolic acidosis.

The main manifestations of the case we reported were irritability, flushing of facial and trunk skin, fast heart rate, breathing fast. These phenomena may involve multiple mechanisms, such as opioid peptides, prostaglandins, histamine, kinin, and catecholamines, which are related to acetaldehyde-activated vasodilation [9].

Although the symptoms of the boy we reported were mild, there are also apparent life-threatening acute ethanol intoxication events. For example, Chang-Teng Wu [10] reported an acute fatal alcohol intoxication incident due to child abuse in a 3-day-old newborn who was fed about 50 ml of rice wine instead of milk by his father. When his father woke up the next day, he found that the neonate had general cyanosis and was not spontaneously breathing. Laboratory tests showed that the child had severe acidosis, abnormal renal and liver function. Unfortunately, after cardiopulmonary–cerebral resuscitation, intermittent mandatory ventilation support and symptomatic treatment, the patient died.

An important reason for the treatment failure of this 3-day-old newborn is that he was not found in time. Consequently, the timely discovery of ethanol intoxication in a neonate is very important for ensuring his health and safety. Fortunately, the neonate in our case was taken to hospital shortly after being found to have accidentally consumed ethanol.

Because the child we reported had a history of ethanol consumption, we immediately gave normal saline gastric lavage until clear fluid was extracted, and diuretic and defecation, in order to promote ethanol excretion, as well as intravenous infusion of naloxone. Naloxone is considered to be an " amethystic agent " [11], which can antagonize acute ethanol intoxication through pharmacokinetic mechanism. One possible mechanism of naloxone antagonizing acute ethanol intoxication and its behavioral effects is to accelerate ethanol metabolism by reversing the redox state disorder of liver nicotinamide adenine dinucleotide (phosphate) couples caused by drugs [12]. Moreover, clinical studies [13, 14] in China have concluded that naloxone has a definite curative effect on acute ethanol intoxication with few side effects, which is a recommended method for treating ethanol intoxication at present. Therefore, we chose to treat the child with intravenous naloxone. And after the above treatments, the symptoms of the child were quickly alleviated.

To sum up, in the grim situation of the global COVID-19 pandemic, the feeding safety of newborns should be taken seriously. The combination of medical history, physical examination, and ethanol poison detection is essential for the timely diagnosis and management of neonatal acute ethanol intoxication. In addition, high concentration ethanol used for inactivating SARS-COV-2 should also be placed reasonably and prominently marked during the COVID-19 epidemic.

## Data Availability

The datasets used and/or analyzed during the current case reports are available from the corresponding author on reasonable request.
